# Supplementary material for: Lipoprotein metabolism mediates hematopoietic stem cell responses under acute anemic conditions
Source: Nat Commun. 2024 Sep 16;15:8131. doi: 10.1038/s41467-024-52509-w (PMC11405780; doi:10.1038/s41467-024-52509-w)
Supplement: Supplementary file 1 — Supplementary Information [file 41467_2024_52509_MOESM1_ESM.pdf]

Supplementary information:

Lipoprotein metabolism mediates hematopoietic stem cell responses under acute anemic conditions

Kiyoka Saito, Mark van der Garde, Terumasa Umemoto, Natsumi Miharada, Julia Sjöberg, Valgardur Sigurdsson, Haruki Shirozu, Shunsuke Kamei, Visnja Radulovic, Mitsuyoshi Suzuki, Satoshi Nakano,Stefan Lang, Jenny Hansson, Martin L Olsson, Takashi Minami, Gunnar Gouras, Johan Flygare, and Kenichi Miharada

Supplementary Figure 1

Supplementary Figure 2

Supplementary Figure 3

Supplementary Figure 4

Supplementary Figure 5

Supplementary Figure 6

Supplementary Data 1.

Supplementary Data 2.

Supplementary Data 3.

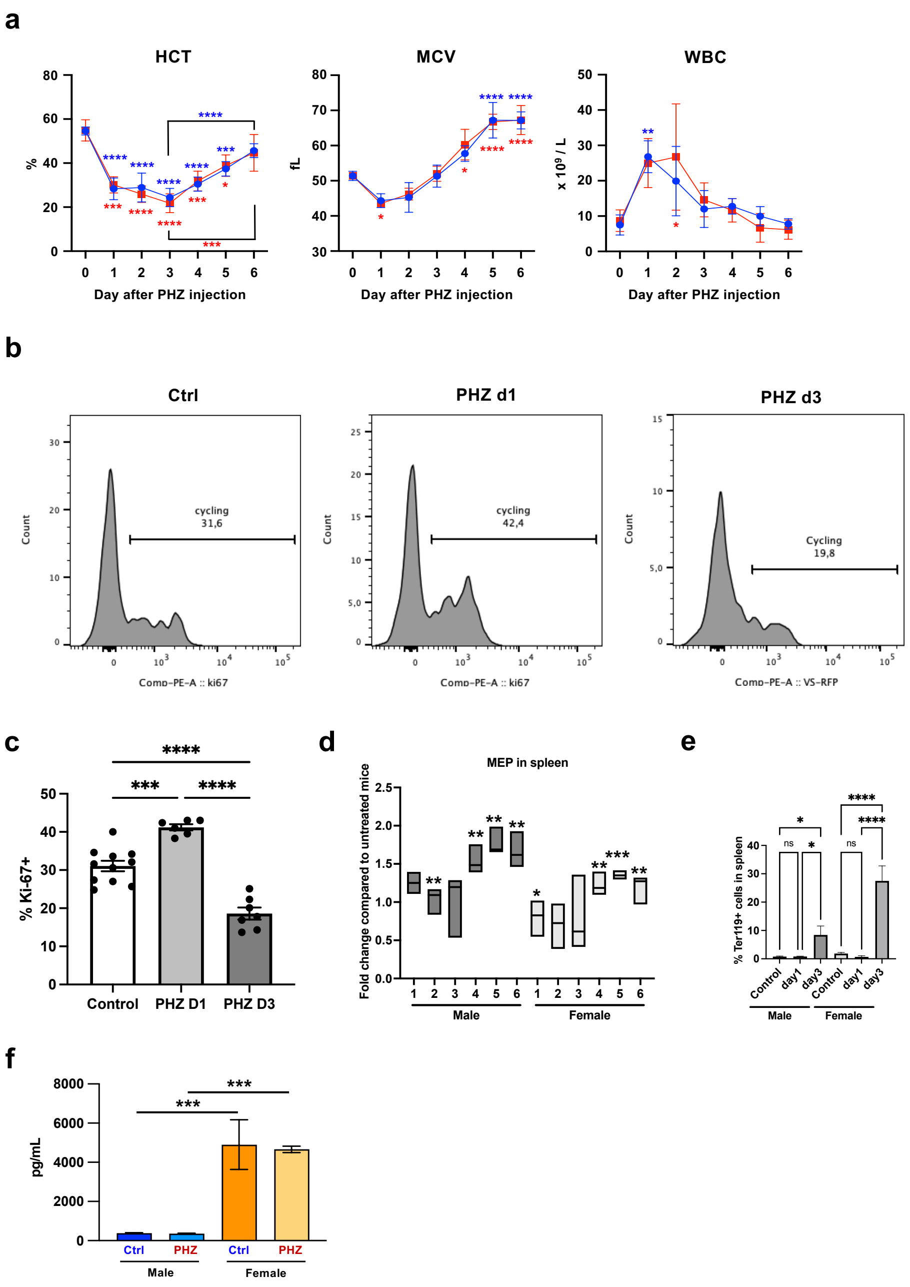

Supplementary Figure 1. Peripheral blood profiles and HSC cell cycle status in PHZ-treated mice

(**a**) PB analysis of the PHZ-treated mice. Hematocrit (HCT), Mean corpuscular volume (MCV), and white blood cell number (WBC) are shown. Please also see Figure 1B. Mean ± SEM are shown. Significance was calculated between day 0 (untreated) and each time point unless separately indicated. n=3-4. Adjusted *p* values were obtained using one-way ANOVA (Tukey's multiple comparison test). (**b-c**) Cell cycle analysis

using Ki-67 expression in HSCs of Ki-67 reporter mice under steady-state condition (Ctrl) and PHZ-treatment (PHZ) (days 1 and 3). Representative Ki-67 histograms gated on $CD150^+CD48^-$KSL cells (**b**) and the mean values of the analysis (**c**) are shown (Mean ± SEM, n=6-11). Adjusted *p* values were obtained using one-way ANOVA (Tukey's multiple comparison test). (**d**) Fold change of MEP populations in male and female spleen. Fold changes compared with day 0 (untreated) mice (mean ± SEM) are shown. n=3-6. *P* values were obtained using one sample t and Wilcoxon test. (**e**) Fold change of $Ter119^+$ cells in male and female spleen. Fold changes compared with day 0 (untreated) mice (mean ± SEM) are shown. n=3-6. Adjusted *p* values were obtained using one-way ANOVA (Tukey's multiple comparison test). (**f**) ELISA assay to measure concentrations of estradiol in blood plasma of male and female mice, treated with or without PHZ. n=4-5. Adjusted *p* values were obtained using one-way ANOVA (Tukey's multiple comparison test).

*$p < 0.05$, **$p < 0.01$, ***$p < 0.001$, ****$p < 0.0001$. Exact *P* values are provided as Source Data.

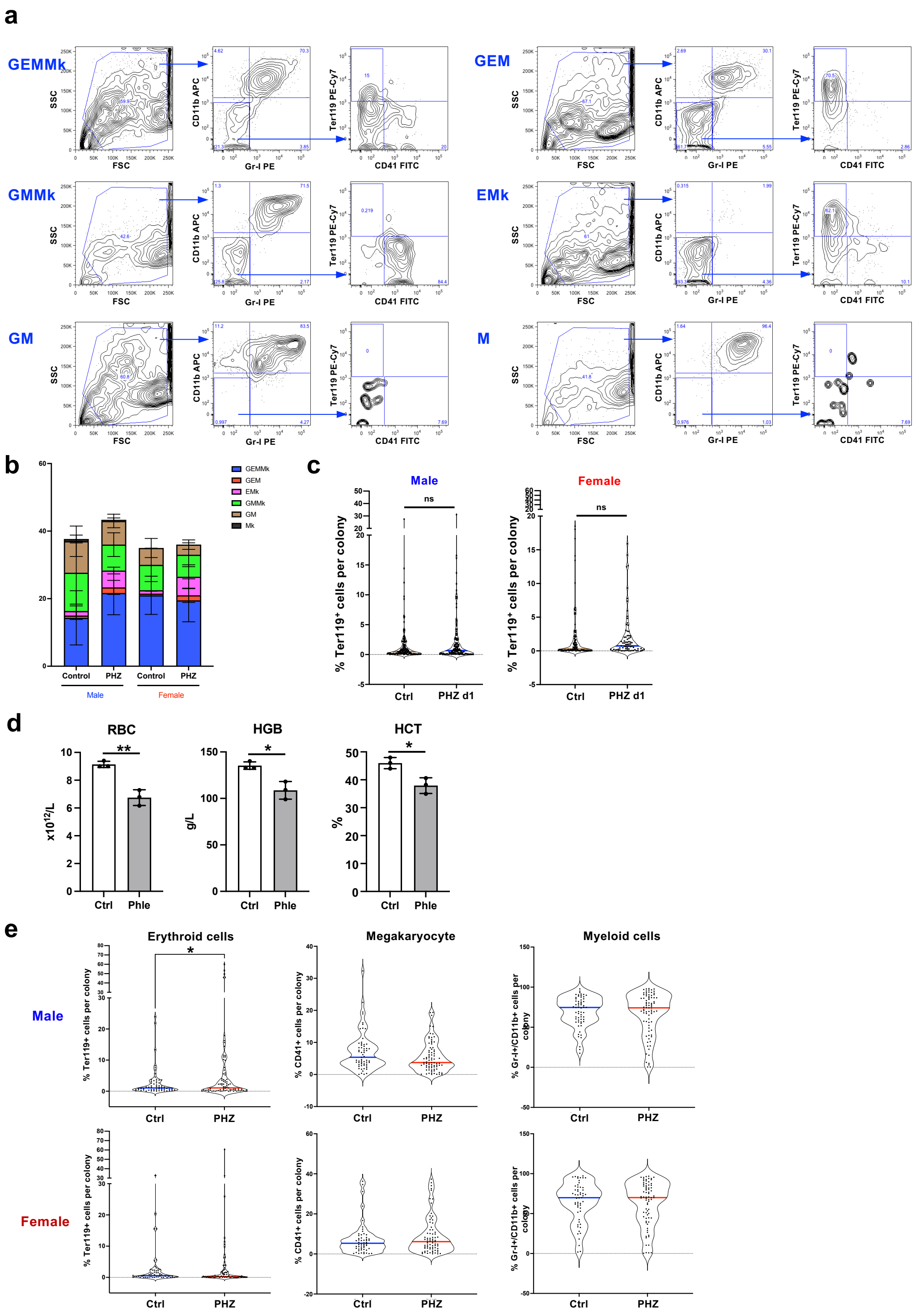

Supplementary Figure 2. Altered lineage balance of HSC-derived colonies (CFU-FACS) upon acute anemia induction.

(**a**) Representative FACS plot of different types of colonies. According to the presence of each lineage, the following types of colonies were identified; Granulocyte-Erythrocyte-Megakaryocyte-Macrophage colony (GEMM), Granulocyte-Erythrocyte-

Macrophage colony (GEM), Granulocyte-Megakaryocyte-Macrophage colony (GMMk), Erythrocyte-Megakaryocyte colony (EMk), Granulocyte-Macrophage colony (GM), and Macrophage colony (M). (**b**) Frequency of different colony types categorized based on CFU-FACS analyses. The existence of erythroid cells and megakaryocytes was determined by detection of more than 1 % Ter119$^+$ cells or 1 % CD41$^+$ cells, respectively. The existence of granulocytes/macrophages was determined by detection of more than 20 % Gr-I$^+$/Mac-I$^+$ cells. n=70-130 from the total of 3 experiments. Adjusted *p* values were obtained using one-way ANOVA (Tukey's multiple comparison test). (**c**) CFU-FACS of HSCs derived from untreated (Ctrl) or PHZ-treated male mice on day 1. *P* values were obtained using Man Whitney tests. (**d**) PB analysis of phlebotomized mice and untreated mice. Red blood cell count (RBC), hemoglobin concentration (HGB), and hematocrit content (HCT) are shown. Mean ± SEM are shown. Significance was calculated between day 0 (untreated) and each time point unless separately indicated. n=4. (**e**) CFU-FACS of HSC derived from the spleen of PBS-control (Ctrl) or PHZ-treated mice. Data of male and female mice are shown. n=55-81 from the total of 3 experiments. *P* values were obtained using unpaired *t* tests. $^*p < 0.05$, $^{**}p < 0.01$, $^{***}p < 0.001$, $^{****}p < 0.0001$. Exact *P* values are provided as Source Data.

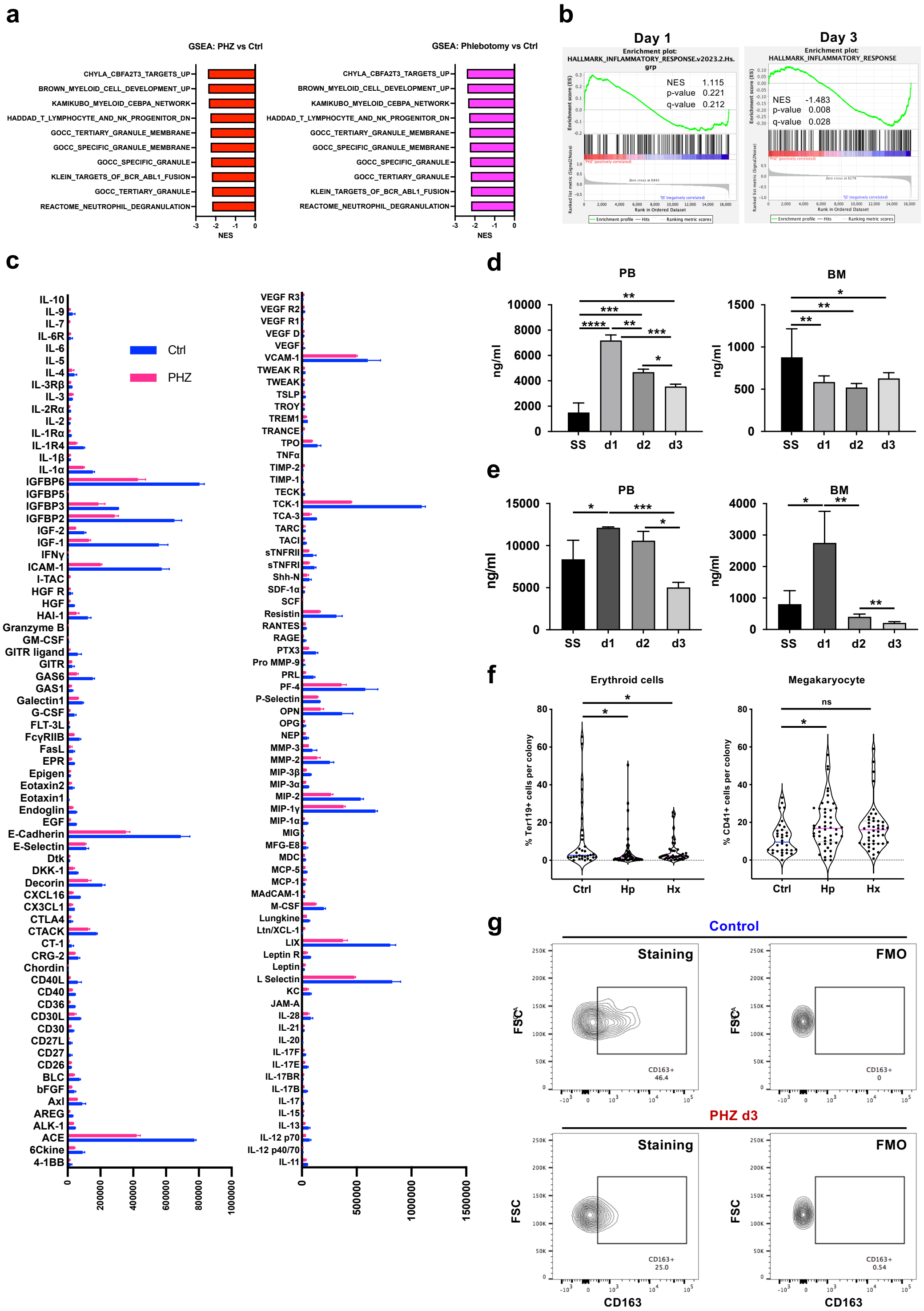

Supplementary Figure 3. Scavenging factors enhance megakaryopoiesis.

(**a**) Summary of GSEA comparing Ctrl vs PHZ and Phle. The top 10 gene signatures are shown. NES: normalized enrichment score. Please see also Supplementary Table S2. (**b**) GSEA data showing no enrichment of unfolded protein response signature in HSCs after PHZ treatment. Left: Day 1, Right: Day 3. (**c**) Cytokine-Array comparing

the abundance of 144 cytokines in the bone marrow fluid of mice between control and PHZ treatment. (**d-e**) ELISA assay to measure concentrations of free hemoglobin (Hb, **d**) and haptoglobin (Hp, **e**) in blood plasma (left) or BM fluid (right) after PHZ treatment. n=3-4. Adjusted *p* values were obtained using one-way ANOVA (Tukey's multiple comparison test). (**f**) CFU-FACS of HSCs treated with recombinant Hp or hemopexin (Hpx). n=32-49 from the total of 3 experiments. Adjusted *p* values were obtained using one-way ANOVA (Tukey's multiple comparison test). (**g**) Representative FACS plot of CD163 expression on $CD150^{+}CD34^{-}$ population (KSL gated) of Ctrl and PHZ-treated mice. FMO: fluorescent minus one control (without CD163 staining).

$^{*}p < 0.05$, $^{**}p < 0.01$, $^{***}p < 0.001$, $^{****}p < 0.0001$. Exact *P* values are provided as Source Data.

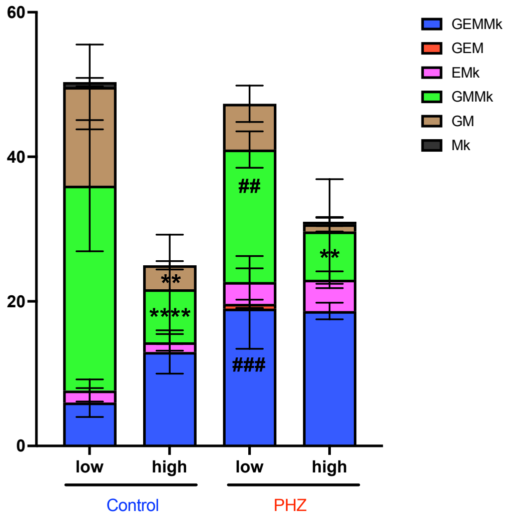

Supplementary Figure 4. VLDLR^{high}HSCs and VLDLR^{low}HSCs have distinct colony formation capacity.

Frequency of different colony types formed from VLDLR^{high}HSCs and VLDLR^{low}HSCs of untreated or PHZ-injected mice, categorized based on CFU-FACS analyses. n=75-151 from the total of 3 experiments. Adjusted *p* values were obtained using one-way ANOVA (Tukey's multiple comparison test).

$^{**}p < 0.01$, $^{****}p < 0.0001$ (compared with VLDLR^{low}HSC in the same mice). $^{\#\#}p < 0.01$, $^{\#\#\#}p < 0.001$ (compared with the same population in the control mice). Exact *P* values are provided as Source Data.

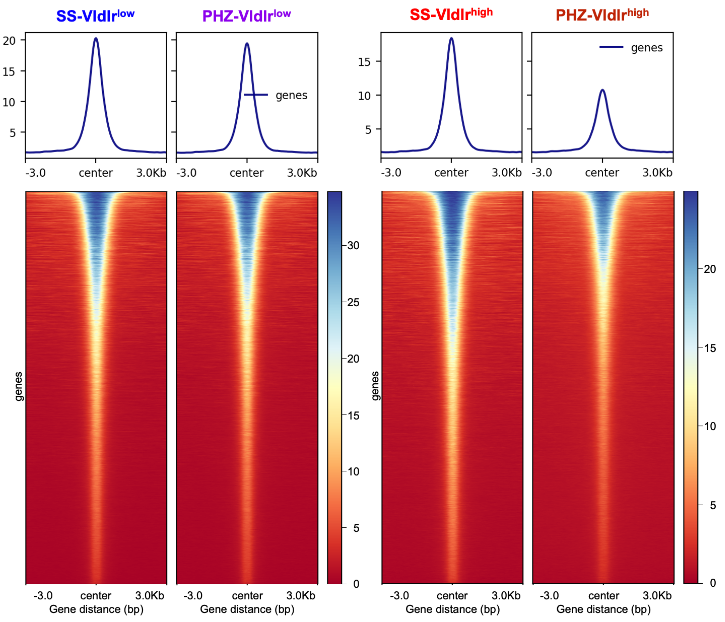

Supplementary Figure 5. PHZ treatment leads to the closing of the chromatin regions in VLDLR^{high}HSCs.

Average plot (top) and heatmap (bottom) of the open chromatin regions which were originally unchanged between VLDLR^{low}HSCs and VLDLR^{high}HSCs under steady-state conditions. Chromatin accessibility in VLDLR^{high} and VLDLR^{low}HSCs are compared between steady state (SS) and PHZ-treated (PHZ) conditions.

a

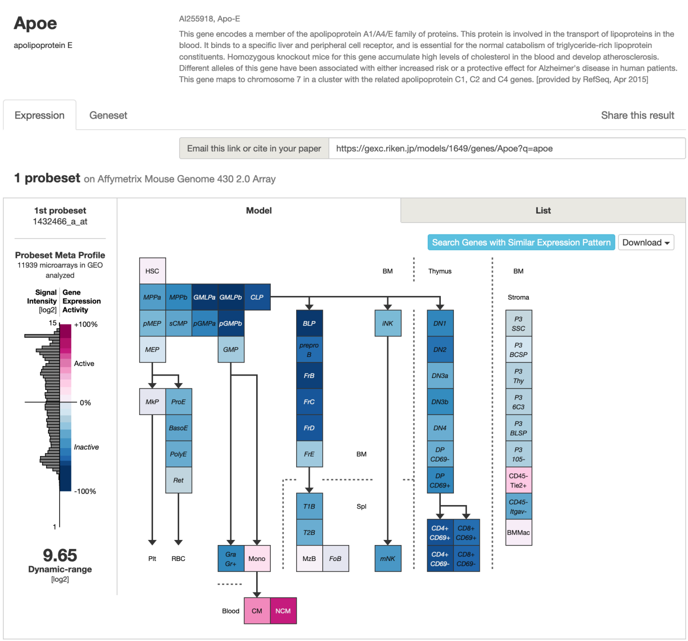

b

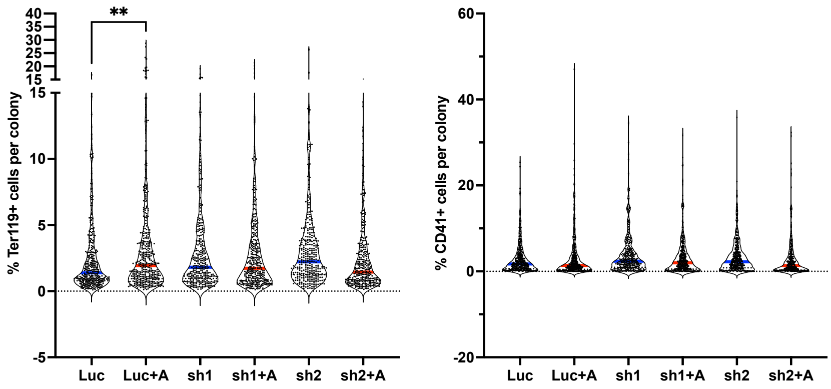

Supplementary Figure 6. Vldlr expression plays a major role in the response of HSC against acute anemia stress

(**a**) Expression pattern of Apoe in various hematopoietic cells. The data from Gene Expression Commons (https://gexc.riken.jp) is shown. (**b**) CFU-FACS of *Vldlr* knocked-down HSCs. WT HSCs were infected with the lentivirus expressing validated shRNA targeting Vldlr or luciferase, and transplanted into lethally irradiated mice. Three months later, the mice were injected with PHZ and CFU-FACS was performed using isolated HSCs. As a result, we observed that Vldlr-knockdown alleviated the increase in erythroid potential upon PHZ injection while shLuciferase control cells still showed higher erythroid content. Adjusted *p* values were obtained using one-way ANOVA

(Tukey's multiple comparison test). *$p < 0.05$, **$p < 0.01$, ***$p < 0.001$, ****$p < 0.0001$.

Exact *P* values are provided as Source Data.
