# Supplementary material for: Lipoprotein metabolism mediates hematopoietic stem cell responses under acute anemic conditions
Source: Nat Commun. 2024 Sep 16;15:8131. doi: 10.1038/s41467-024-52509-w (PMC11405780; doi:10.1038/s41467-024-52509-w)
Supplement: Supplementary file 3 — Description Of Additional Supplementary File [file 41467_2024_52509_MOESM3_ESM.pdf]

**Description of Additional supplementary file**

**Supplementary Data 1**: List of genes up- or downregulated upon PHZ injection or phlebotomy.

**Supplementary Data 2**: Summary of GSEA comparing PHZ and Phle vs Ctrl at day 3.

**Supplementary Data 3**: Summary of GSEA comparing PHZ vs Ctrl HSC at day 1.
