# Supplementary material for: Lipoprotein metabolism mediates hematopoietic stem cell responses under acute anemic conditions
Source: Nat Commun. 2024 Sep 16;15:8131. doi: 10.1038/s41467-024-52509-w (PMC11405780; doi:10.1038/s41467-024-52509-w)
Supplement: Supplementary file 7 — Reporting summary [file 41467_2024_52509_MOESM7_ESM.pdf]

nature portfolio

Corresponding author(s): Kenichi Miharada

Last updated by author(s): Aug 7, 2024

Reporting Summary

Nature Portfolio wishes to improve the reproducibility of the work that we publish. This form provides structure for consistency and transparency in reporting. For further information on Nature Portfolio policies, see our Editorial Policies and the Editorial Policy Checklist.

Statistics

For all statistical analyses, confirm that the following items are present in the figure legend, table legend, main text, or Methods section.

n/a	Confirmed	
☐	☒	The exact sample size (*n*) for each experimental group/condition, given as a discrete number and unit of measurement
☐	☒	A statement on whether measurements were taken from distinct samples or whether the same sample was measured repeatedly
☐	☒	The statistical test(s) used AND whether they are one- or two-sided<br>*Only common tests should be described solely by name; describe more complex techniques in the Methods section.*
☒	☐	A description of all covariates tested
☒	☐	A description of any assumptions or corrections, such as tests of normality and adjustment for multiple comparisons
☐	☒	A full description of the statistical parameters including central tendency (e.g. means) or other basic estimates (e.g. regression coefficient) AND variation (e.g. standard deviation) or associated estimates of uncertainty (e.g. confidence intervals)
☐	☒	For null hypothesis testing, the test statistic (e.g. F, t, r) with confidence intervals, effect sizes, degrees of freedom and P value noted<br>*Give P values as exact values whenever suitable.*
☒	☐	For Bayesian analysis, information on the choice of priors and Markov chain Monte Carlo settings
☒	☐	For hierarchical and complex designs, identification of the appropriate level for tests and full reporting of outcomes
☒	☐	Estimates of effect sizes (e.g. Cohen's d, Pearson's r), indicating how they were calculated

Our web collection on statistics for biologists contains articles on many of the points above.

Software and code

Policy information about availability of computer code

Data collection	N/A
Data analysis	Microarray data was analyzed in R (version 3.5.0) using R studios (version 1.2.5019), heatmaps were generated using the pheatmap package (version 1.0.10). The data was also analyzed using Gene Set Enrichment Analysis (GSEA, version 4.0.0), and the results were redrawn using the replotGSEA function from the Rtoolbox package. All statistical analyses were done using Prism (version 9, GraphPad).

For manuscripts utilizing custom algorithms or software that are central to the research but not yet described in published literature, software must be made available to editors and reviewers. We strongly encourage code deposition in a community repository (e.g. GitHub). See the Nature Portfolio guidelines for submitting code & software for further information.

Data

Policy information about availability of data

All manuscripts must include a data availability statement. This statement should provide the following information, where applicable:

- Accession codes, unique identifiers, or web links for publicly available datasets
- A description of any restrictions on data availability
- For clinical datasets or third party data, please ensure that the statement adheres to our policy

The microarray data generated in this study have been deposited in the GEO database under accession codes GSE162408 and GSE212392. The ATAC-seq data generated in this study have been deposited in the GEO database under accession code GSE274144.

Research involving human participants, their data, or biological material

Policy information about studies with human participants or human data. See also policy information about sex, gender (identity/presentation), and sexual orientation and race, ethnicity and racism.

Reporting on sex and gender	N/A
Reporting on race, ethnicity, or other socially relevant groupings	N/A
Population characteristics	N/A
Recruitment	N/A
Ethics oversight	N/A

Note that full information on the approval of the study protocol must also be provided in the manuscript.

Field-specific reporting

Please select the one below that is the best fit for your research. If you are not sure, read the appropriate sections before making your selection.

☒ Life sciences ☐ Behavioural & social sciences ☐ Ecological, evolutionary & environmental sciences

For a reference copy of the document with all sections, see nature.com/documents/nr-reporting-summary-flat.pdf

Life sciences study design

All studies must disclose on these points even when the disclosure is negative.

Sample size	No prospective sample-size calculations were performed. Sample size was determined retrospectively to be adequate on the basis of the consistency and magnitude
Data exclusions	No data was excluded
Replication	All experiments were repeated more than three times to verify the results. For CRISPR and shRNA-related experiments multiple clones (both successful and failed editing/down-regulation were expected) were included in the assay in order to confirm the consistency.
Randomization	The chemical compound screening was performed using pre-deposited plates. In order to false signals based on the "edge effect" in which specific loci of wells in plastic plates are affected by unintended factors (e.g. evaporation and reflection of fluorescence), control assay were performed to compare fluorescent signals between random positions.
Blinding	Scoring of enucleated cells on cytospin slides were performed without showing the sample labelling, as a blind test.

Reporting for specific materials, systems and methods

We require information from authors about some types of materials, experimental systems and methods used in many studies. Here, indicate whether each material, system or method listed is relevant to your study. If you are not sure if a list item applies to your research, read the appropriate section before selecting a response.

Materials & experimental systems

n/a	Involved in the study	
☐	☒	Antibodies
☒	☐	Eukaryotic cell lines
☒	☐	Palaeontology and archaeology
☐	☒	Animals and other organisms
☒	☐	Clinical data
☒	☐	Dual use research of concern
☒	☐	Plants

Methods

n/a	Involved in the study	
☒	☐	ChIP-seq
☐	☒	Flow cytometry
☒	☐	MRI-based neuroimaging

Antibodies

Antibodies used	anti-Vldlr (Abcam), -CD163 (TNKUPJ, eBiosciences), -CD34 (RAM34, BD), -CD150 (TC15-12F12.2, BioLegend), -CD16/32 (93,

Antibodies used	BioLegend), -c-Kit (2B8, eBioscience), -Sca-1 (D7, BioLegend), -CD127 (A7R34, BioLegend), -CD45.1 (A20, BioLegend), -CD45.2 (104, BioLegend), -CD3 (145-2C11, BioLegend), -CD4 (H129.19, BD), -CD8 (53-6.7, BioLegend), -Gr-1 (RB6-8C5, BioLegend), -CD11b (M1/70, BioLegend), -B220 (RA3-6B2, BioLegend), and Ter119 (TER119, BioLegend).
Validation	All antibodies have been used in previous publications.

Animals and other research organisms

Policy information about studies involving animals; ARRIVE guidelines recommended for reporting animal research, and Sex and Gender in Research

Laboratory animals	Young C57BL/6J (Ly-5.2) male mice were obtained from Janvier Labs. All animals were maintained in individually ventilated racks and given autoclaved food and water ad libitum. Kusabira Orange (KuO) mice were gifted from Hiromitsu Nakauchi's laboratory at Tokyo University, Japan. ApoE knockout mice (B6.129P2-Apoetm1Unc/J) and Ki-67 reporter mice (Mki67tm1.1Cle/J)72 were purchased from The Jackson Laboratory.
Wild animals	N/A
Reporting on sex	We compared the data between male and female mice, and they are shown in the figures
Field-collected samples	N/A
Ethics oversight	All experiments were approved by the Lund University Animal Ethical Committee, Swedish Board of Agriculture, and Animal Research Facility of Kumamoto University guidelines.

Note that full information on the approval of the study protocol must also be provided in the manuscript.

Plants

Seed stocks	N/A
Novel plant genotypes	N/A
Authentication	N/A

Flow Cytometry

Plots

Confirm that:

☒ The axis labels state the marker and fluorochrome used (e.g. CD4-FITC).

☒ The axis scales are clearly visible. Include numbers along axes only for bottom left plot of group (a 'group' is an analysis of identical markers).

☒ All plots are contour plots with outliers or pseudocolor plots.

☒ A numerical value for number of cells or percentage (with statistics) is provided.

Methodology

Sample preparation	Adult BM cells were isolated by crushing tibias, femurs, and iliac bones of 8 to 10 weeks old mice with a mortar and pestle in PBS. Mononuclear cells were collected from the buffy coat after Lymphoprep™ separation. C-Kit positive (c-Kit+) cells were enriched using a magnetic separation system (MACS) with anti-c-Kit magnetic beads (Miltenyi Biotec). The enriched cells were stained with antibodies (mentioned above) conjugated to FITC, PE, PE-Cy5, PE-Cy7, APC, APC-eFluor780 or Brilliant Violet:
Instrument	Cells were sorted on FACS Aria III or analyzed on FACS LSRII or LSR Fortessa (BD).
Software	Collected data were analyzed on the FlowJo software (Tree Star).
Cell population abundance	Since hematopoietic stem cells are a rare population, it is not realistic to check the purity of sorted cells all the time. However we checked sorting efficiency as a pilot experiment.

Gating strategy

We always prepared FMO (fluorescent minus one) control to decide all gatings

☒ Tick this box to confirm that a figure exemplifying the gating strategy is provided in the Supplementary Information.
